# Supplementary material for: FDG-PET/CT Versus Contrast-Enhanced CT for Response Evaluation in Metastatic Breast Cancer: A Systematic Review
Source: Diagnostics (Basel). 2019 Aug 27;9(3):106. doi: 10.3390/diagnostics9030106 (PMC6787711; doi:10.3390/diagnostics9030106)
Supplement: Supplementary file 1 [file diagnostics-09-00106-s001.pdf]

Supplementary content 1: Full search strategies for PubMed/Medline, Cochrane library, and Embase (conducted on 10th October 2017).

Pubmed/Medline and Cochrane library:

((breast cancer[MeSH Terms]) OR breast tumor) OR breast tumor*) OR breast carcinoma) OR breast neoplasms) OR breast neoplasm*) OR cancer of the breast*) OR cancer mamma*) OR mamma* cancer) OR malignant neoplasia of the breast*) OR breast malignant neoplasm*) OR breast malignant tumor*) OR breast carcinoma*) OR human mammary carcinoma*) OR human mammary neoplasm*) OR breast sarcoma) OR breast sarcoma*) OR mammary sarcoma*))
AND
((neoplasm metastases[MeSH Terms]) OR neoplasm metastasis[MeSH Terms]) OR distant metastasis) OR metastasis) OR satellite metastasis) OR metasta*) OR disseminat*) OR secondary) OR migrat*) OR stage 4) OR stage IV) OR stage four) OR advanced cancer) OR advanced)))
AND
(RECIST) OR response evaluation criteria in solid tumors) OR PERCIST) OR PET response criteria in solid tumors)

Embase (results shown are from an identical search on 19 December 2017, hence more hits than stated in the article):

265	exp breast cancer/	398263	Advanced	Display Results | More
266	exp breast tumor/ or breast tumor*.mp.	467308	Advanced	Display Results | More
267	exp breast carcinoma/ or exp breast neoplasms/ or breast neoplasm*.mp.	471313	Advanced	Display Results | More
268	cancer of the breast*.mp.	19070	Advanced	Display Results | More
269	cancer mamma*.mp.	304	Advanced	Display Results | More
270	mamma* cancer.mp.	3970	Advanced	Display Results | More
271	malignant neoplasia of the breast*.mp.	7	Advanced	Display Results | More
272	breast malignant neoplasm*.mp.	11	Advanced	Display Results | More
273	breast malignant tumor*.mp.	46	Advanced	Display Results | More
274	breast carcinoma*.mp.	79193	Advanced	Display Results | More
275	human mammary carcinoma*.mp.	883	Advanced	Display Results | More
276	human mammary neoplasm*.mp.	3	Advanced	Display Results | More
277	exp breast sarcoma/ or breast sarcoma*.mp.	1016	Advanced	Display Results | More
278	mammary sarcoma*.mp.	98	Advanced	Display Results | More
279	265 or 266 or 267 or 268 or 269 or 270 or 271 or 272 or 273 or 274 or 275 or 276 or 277 or 278	480304	Advanced	Display Results | More
280	exp distant metastasis/ or metastasis.mp. or exp metastasis/ or exp satellite metastasis/	614701	Advanced	Display Results | More
281	metasta*.mp.	749790	Advanced	Display Results | More
282	disseminat*.mp.	166846	Advanced	Display Results | More
283	secondary.mp.	817807	Advanced	Display Results | More
284	migrat*.mp.	407042	Advanced	Display Results | More
285	stage 4.mp.	9046	Advanced	Display Results | More
286	Stage IV.mp.	32287	Advanced	Display Results | More
287	RECIST.mp.	13131	Advanced	Display Results | More
288	Response evaluation criteria in solid tumors.mp. or exp response evaluation criteria in solid tumors/	6071	Advanced	Display Results | More
289	PERCIST.mp.	409	Advanced	Display Results | More
290	PET response criteria in solid tumors.mp.	105	Advanced	Display Results | More
291	265 or 266 or 267 or 268 or 269 or 270 or 271 or 272 or 273 or 274 or 275 or 276 or 277 or 278	480304	Advanced	Display Results | More
292	280 or 281 or 282 or 283 or 284 or 285 or 286	2048951	Advanced	Display Results | More
293	291 and 292	137172	Advanced	Display Results | More
294	287 or 288 or 289 or 290	15178	Advanced	Display Results | More
295	293 and 294	1150	Advanced	Display Results | More
296	exp advanced cancer/ or advanced.mp.	531665	Advanced	Display Results | More
297	stage four.mp.	340	Advanced	Display Results | More
298	280 or 281 or 282 or 283 or 284 or 285 or 286 or 296 or 297	2451792	Advanced	Display Results | More
299	291 and 298	153306	Advanced	Display Results | More
300	294 and 299	1372	Advanced	Display Results | More
